# Supplementary material for: Full-frame and high-contrast smart windows from halide-exchanged perovskites
Source: Nat Commun. 2021 Jun 7;12:3360. doi: 10.1038/s41467-021-23701-z (PMC8184980; doi:10.1038/s41467-021-23701-z)
Supplement: Supplementary file 1 — Supplementary Information [file 41467_2021_23701_MOESM1_ESM.pdf]

Supplementary Information

Full-frame and high-contrast smart windows from halide-exchanged perovskites

You Liu[1]†, Jungan Wang[1]†, Fangfang Wang[1]†, Zhengchun Cheng[1], Yinyu Fang[1], Qing Chang[1], Jixin Zhu[1], Lin Wang[1], Jianpu Wang[1], Wei Huang[2,3]*, Tianshi Qin[1]*

[1]Key Laboratory of Flexible Electronics (KLOFE) & Institution of Advanced Materials (IAM), Jiangsu National Synergetic Innovation Center for Advanced Materials (SICAM), Nanjing Tech University (NanjingTech), Nanjing, Jiangsu 211816, China
[2]Key Laboratory for Organic Electronics & Information Displays (KLOEID) & Institute of Advanced Materials (IAM), Nanjing University of Posts and Telecommunications, Nanjing, Jiangsu 210023, China
[3]Frontiers Science Center for Flexible Electronics & Institute of Flexible Electronics (IFE), Northwestern Polytechnical University (NPU), Xi'an, Shaanxi 710072, China

*Correspondence to: iamtsqin@njtech.edu.cn, iamwhuang@nwpu.edu.cn

†These authors contributed equally to this work.

This supplementary information includes:

Supplementary Figures. 1-10

Supplementary Tables 1-3

Supplementary References (1-17)

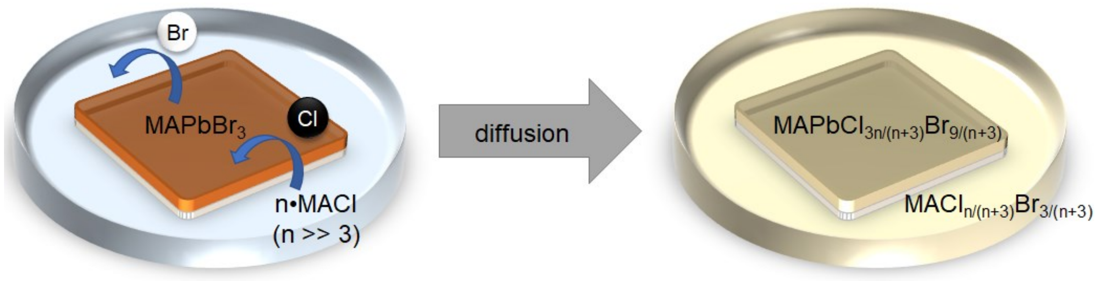

Supplementary Figure 1. Schematic diagram of ion exchange technology. Cl ions in MACl solution are diffusing into $MAPbBr_3$ perovskite film and replacing Br ions in perovskite crystal lattice, resulting in homogenized Br/Cl distribution ($Cl_{n/(n+3)}$:$Br_{3/(n+3)}$) in whole solid/liquid environment. The perovskite film will convert into $MAPbCl_3$, if n>>3.

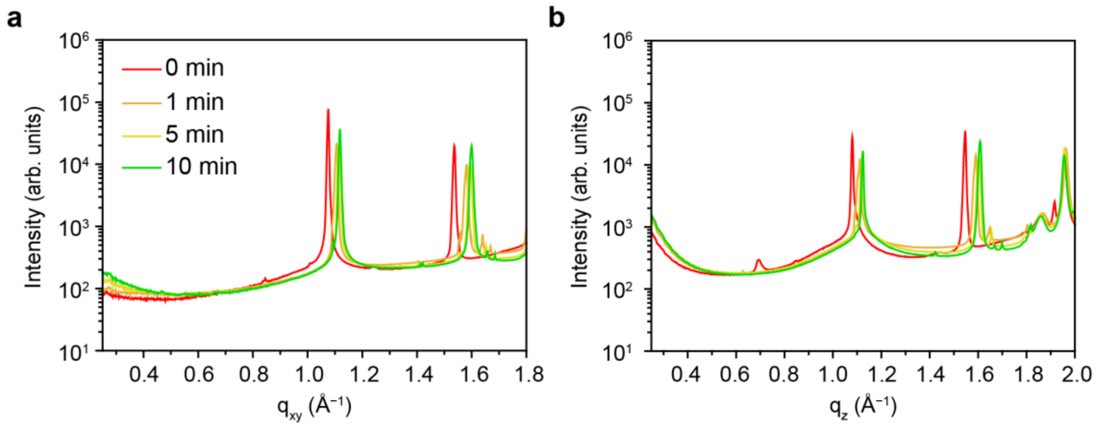

Supplementary Figure 2. GIWAXS 1D Scattering Profiles. a, in-plane and **b**, out-of-plane 1D plots of perovskite films fabricated by variable halide-exchange duration times.

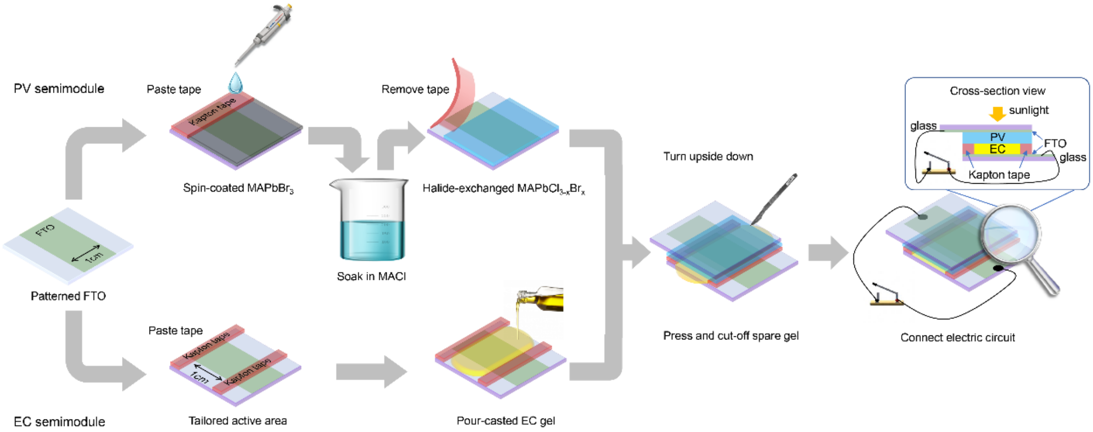

Supplementary Figure 3. Full-solution processes of PV and EC semi-modules, integrating into a monolithic PVCD. Initially compact/mesoporous titanium oxide (TiO_2) as an electron-transporting layer (ETL) and subsequently methylammonium lead bromide ($MAPbBr_3$) perovskite were spin-coated on a patterned fluorine-doped tin oxide (FTO) coated glass substrate, successively. The orange-coloured $MAPbBr_3$ film was then dipped into a methylammonium chloride (MACl) solution for halide-exchanging procedure, resulting in mixed Cl/Br halide perovskite ($MAPbBr_xCl_{3-x}$) with excellent visible-light transmittance. The hole-transporting layer (HTL) was subsequently spin-coated on the top of the perovskite layer, achieving the PV semi-module. On the other side, a flexible electrochromic (EC) ion-gel was pour-casting on another FTO/glass substrate as the EC semi-module. In final two semi-modules were oppositely pressed to form a good ohmic contact, achieving a monolithic photovoltachromic device (PVCD), in which the size and thickness were adjusted by overlapped FTO area and Kapton tape height.

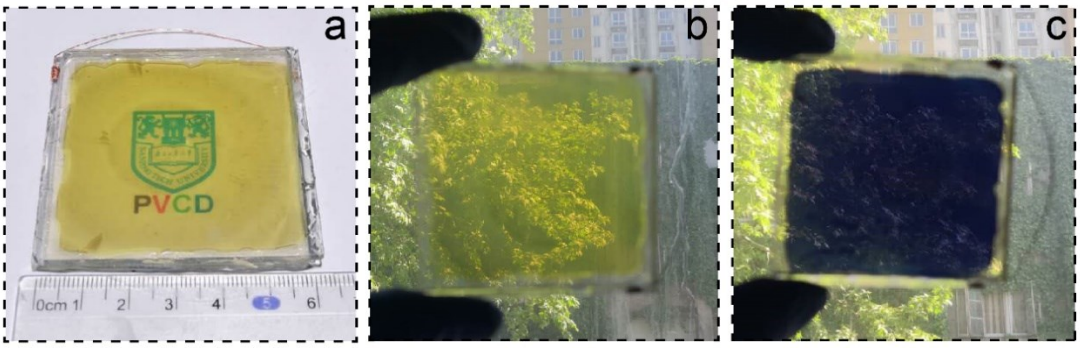

Supplementary Figure 4. Photographs of PVCDs. a, Large PVCD with active area of 36 cm^2 (6 cm × 6 cm). **b**, Bleaching state and **c**, Colouring state of large PVCD.

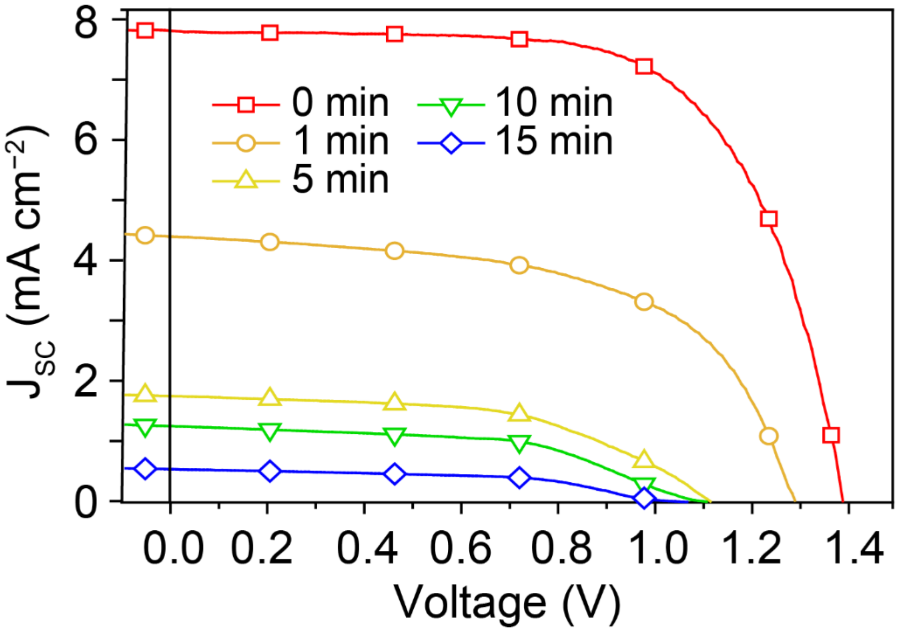

Supplementary Figure 5. The *J-V* curves of PV devices fabricated by variable halide-exchange duration times.

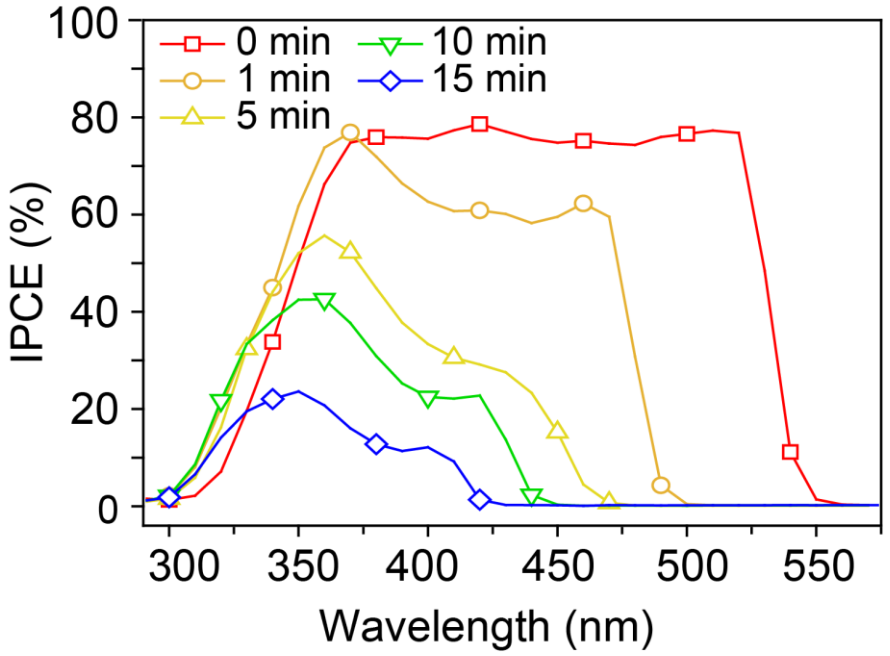

Supplementary Figure 6. Photoresponse of PV. IPCE curves of PV devices fabricated by variable halide-exchange duration times from 300 to 575 nm wavelengths.

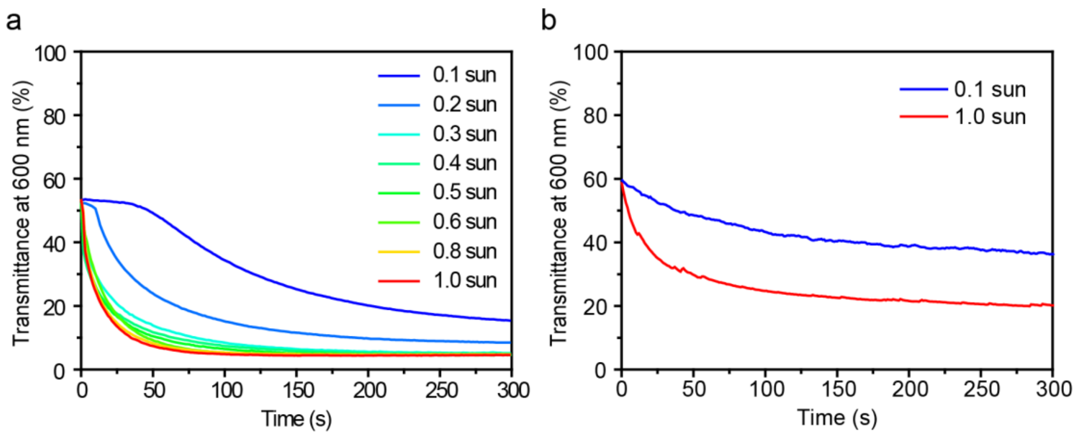

Supplementary Figure 7. Real-time transmission tendencies at 600 nm. a, PVCD based on $MAPbBr_3$ and **b,** photochromic lens illuminated under variable solar irradiations between 0.1 and 1.0 sun.

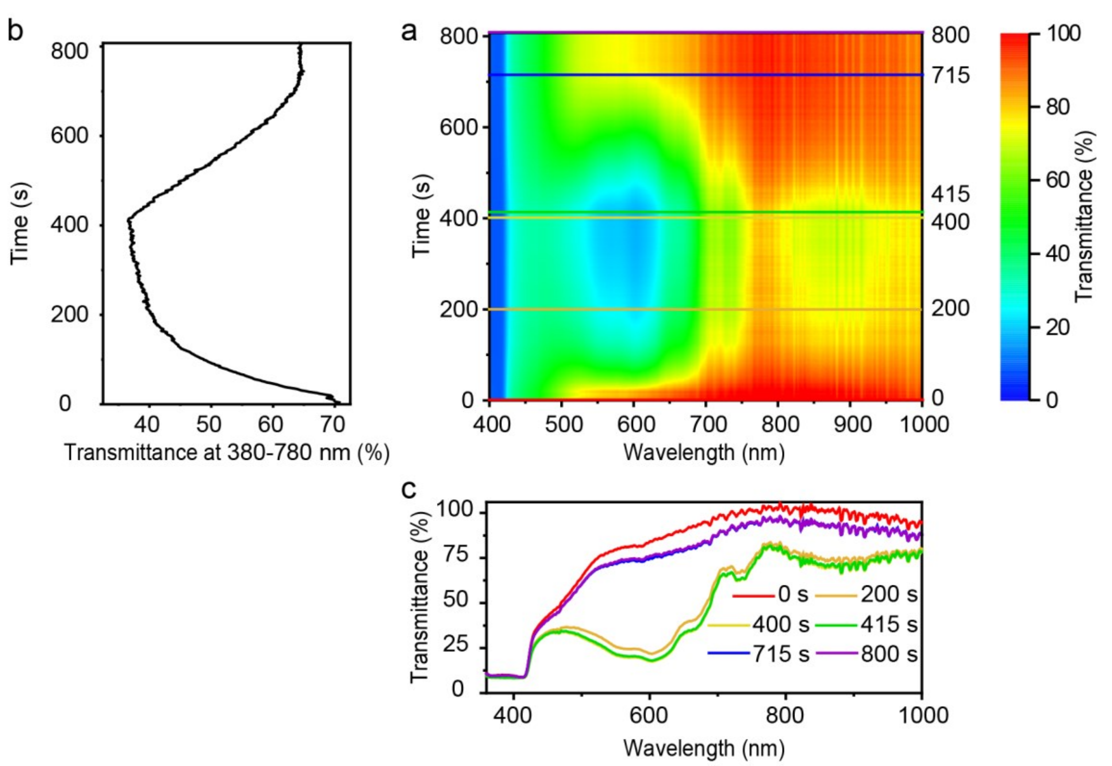

Supplementary Figure 8. Optical properties of PVCD. a, Real-time dynamic visible and infrared transmittance spectra of the PVCDs were investigated under 1.0 sun illumination. **b,** Real-time AVT (380-780 nm) spectra of the PVCD under connection (0-415 s) and disconnection (415-800 s). **c,** Full-wavelength (380-1000 nm) transmittance spectra of the PVCD illuminated under 1.0 sun at 0, 200, 400, 415, 715 and 800th s, respectively.

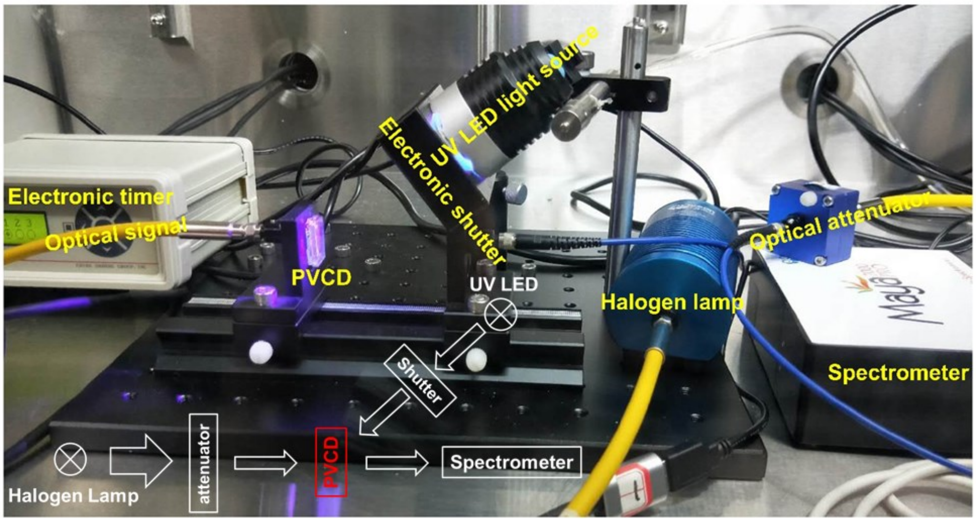

Supplementary Figure 9. Schematic diagram of the in-situ optical characteristic system. Spectrometer: Maya 2000, VIS-NIR, 360-1100 nm, Ocean Optics; Halogen light source: HL-2000, Ocean Optics; Optical attenuator: FVA-UV, 200-2500 nm, Wenyi Optics, China; Electronic shutter and timer: GCI-73M, Φ 20 mm, Daheng Optics, China.

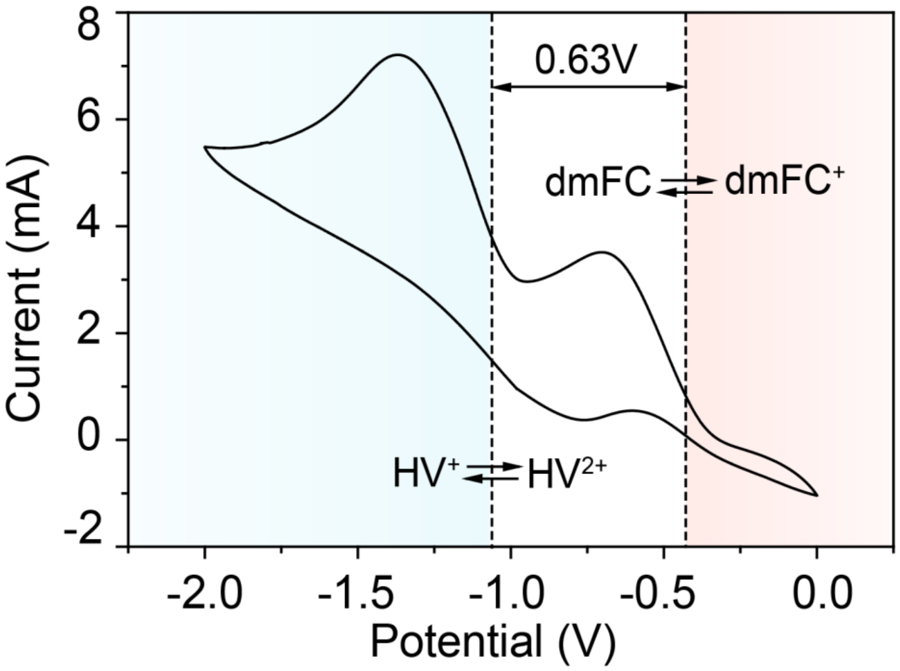

Supplementary Figure 10. Cyclic voltammogram curve of oxidant and reductant pair in EC gel, indicating the driving voltage for EC at ca. 0.63 V.

Supplementary Table 1. Comparison of transmittance performance of semi-transparent PV films prepared by various preparation methods.

Device photograph	Materials	Preparation method	Transmittance performance (AVT %)	References
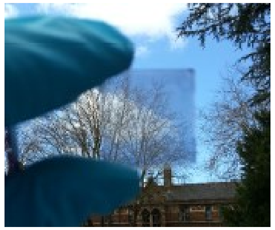	$CH_3NH_3PbI_{3-x}Cl_x$	with a voids pattern and chemically broadening the optical bandgap	50% (370-740 nm)	*ACS Nano* **2014**, 8, 1, 591.[1]
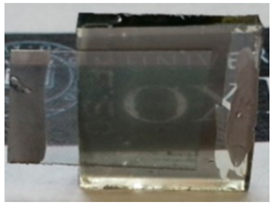	$CH(NH_2)_2PbI_3$ and $CH_3NH_3PbI_3$	Physical void pattern	30% (370-740 nm)	*J. Phys. Chem. Lett.* **2015**, 6, 129.[2]
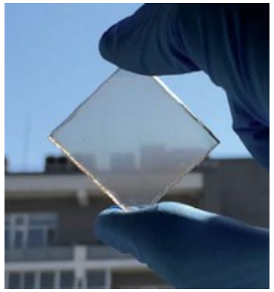	$CH_3NH_3PbI_{3-x}(SCN)_x$	Physical void pattern	50% (380-740 nm)	*Sustainable Energy Fuels,* **2017**, 1, 1034.[3]

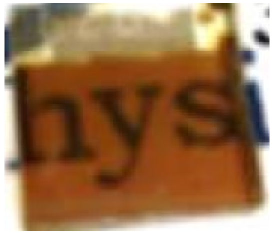	$CH_3NH_3PbI_3$	Physical void pattern	38% (400-750 nm)	*Solar Energy Materials & Solar Cells,* **2017**, 160, 193.[4]
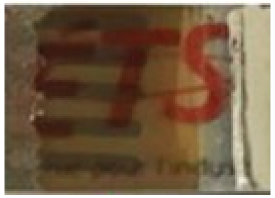	$CH_3NH_3PbI_{3-x}Cl_x$ and $CH_3NH_3PbI_{3-x-y}Cl_xBr_y$	Physical void pattern	25% (400-800 nm)	*Small* **2018**, 14, 1802319.[5]
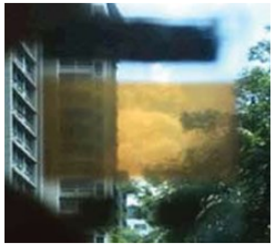	$Cs_{0.2}FA_{0.8}Pb(I_{0.6}Br_{0.4})_3$	Physical void pattern	36% (400-800 nm)	*J. Mater. Chem. A,* **2018**, 6, 23787.[6]
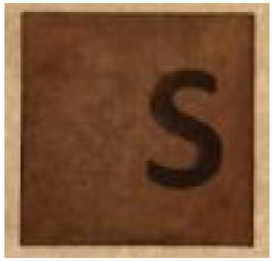	$CH_3NH_3PbI_3$	Physical void pattern	16% (370-740 nm)	*ACS Appl. Mater. Interfaces* **2019**, 11, 10021.[7]

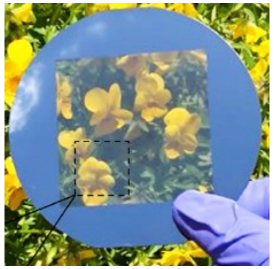	Crystalline silicon	Physical void pattern	50% (300-900 nm)	*Joule* **2020**, 4, 235.[8]
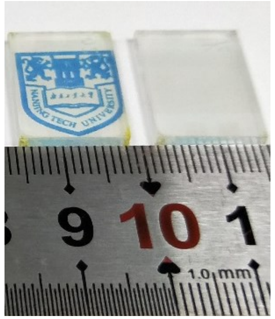	**$CH_3NH_3PbCl_{3-x}Br_x$**	Chemically broadening the optical bandgap	**76% (400-780 nm)**	**Our work**

Supplementary Table 2. Comparison of drawbacks of various device type PECD or PVCD.

Device photograph [a]	Electrolyte phase	Integration type	Electrode amount	Active area ratio	PV transmittance %	Cyclic reversibility	Cyclic stability	References
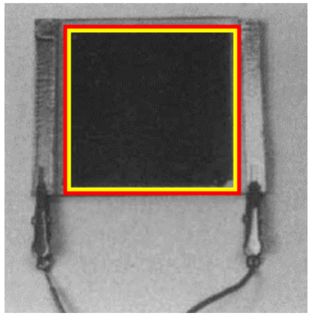	Liquid	Monolithic	2-terminal	100%	~71% (at 788 nm)	N/A	N/A	*Nature*, **1996**, 383, 608–610.[9]
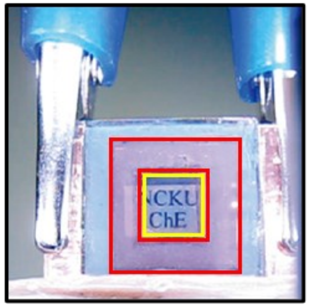	Liquid	Side-by-side	2- or 3-terminal	35%	~60% (at 788 nm)	200 cycles driven by illumination	~100%	*ACS Nano*, **2009**, 3, 2297–2303.[10]

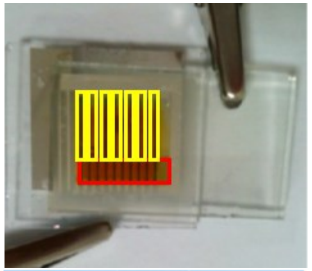	Liquid	Side-by-side	2- or 3-terminal	<50%	~52% (at 650 nm)	3 cycles driven by illumination	~100%	*ACS Appl. Mater. Interfaces*, **2014**, 6, 2415.[11]
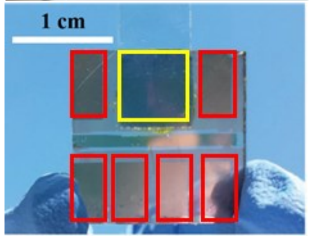	Solid	Side-by-side	4-terminal	25%	26% AVT (370-740 nm)	10^4 cycles driven by electricity	~50%	*Energy Environ. Sci.*, **2015**, 8, 1578.[12]
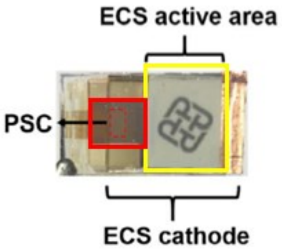	Solid	Side-by-side	3- or 4-terminal	50%	N/A	N/A	N/A	*ACS Nano*, **2016**, 10, 5900.[13]
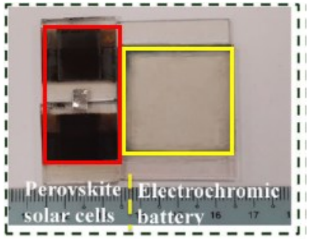	Solid	Side-by-side	4-terminal	50%	~43% (at 750 nm)	2500 cycles driven by electricity	retention of 86.7%	*Mater. Horiz.*, **2016**, 3, 588.[14]

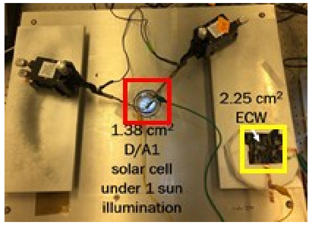	Solid	External circuit	4-terminal	N/A	42% (at 650 nm)	N/A	N/A	*Nat. Energy*, **2017**, 2:17104.[15]
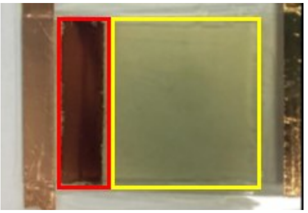	Solid	Side-by-side	2-terminal	80%	~63% (at 529 nm)	10^3 cycles driven by electricity	~75%	*Materials*, **2020**, 13, 1206.[16]
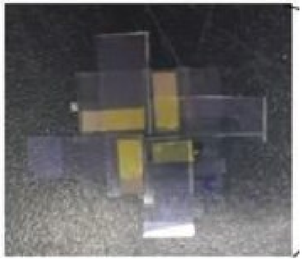	Solid	Tandem	3-terminal	N/A	N/A	10^3 cycles driven by electricity	~89%	*Adv. Funct. Mater*, **2020**, 30, 1909601.[17]
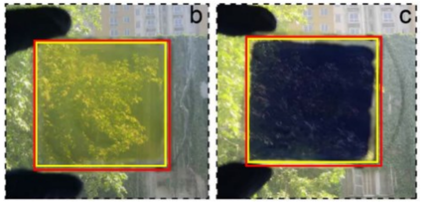	**Solid**	**Monolithic**	**2-terminal**	**100%**	**76% AVT (400-780 nm)**	**10^4 cycles driven by illumination**	**~67%**	**Our work**

[a] In photographs, red square is PV component area and yellow square is EC component area.

Supplementary Table 3. Values of the parameters of the photovoltaic cell fabricated by variable halide-diffusion periods.

Reaction time (min)	V_{OC}(V)	J_{SC} (mA/cm^2)	FF (%)	PCE (%)	
				Best	Average
0	1.38	7.81	66.25	7.16	6.98
1	1.29	4.39	57.15	3.24	3.03
5	1.16	1.84	48.29	1.03	0.99
10	1.10	1.24	51.71	0.71	0.63
15	1.04	0.53	50.80	0.28	0.26

Supplementary References:

1. Eperon, G. E., Burlakov, V. M., Goriely, A. & Snaith, H. J. Neutral Color Semitransparent Microstructured Perovskite Solar Cells. *ACS Nano* **8**, 591-598 (2014).
2. Eperon, G. E. *et al.* Efficient, Semitransparent Neutral-Colored Solar Cells Based on Microstructured Formamidinium Lead Trihalide Perovskite. *J. Phys. Chem. Lett.* **6**, 129-138 (2015).
3. Zhang, L., Hörantner, M. T., Zhang, W., Yan, Q. & Snaith, H. J. Near-neutral-colored semitransparent perovskite films using a combination of colloidal self-assembly and plasma etching. *Sol. Energy Mater. Sol. Cells* **160**, 193-202 (2017).
4. Chen, S. *et al.* Neutral-colored semitransparent solar cells based on pseudohalide (SCN^{-})-doped perovskite. *Sustainable Energy Fuels* **1**, 1034-1040 (2017).
5. Rai, M. *et al.* Hot dipping post treatment for improved efficiency in micro patterned semi-transparent perovskite solar cells. *J. Mater. Chem. A* **6**, 23787-23796 (2018).
6. Ka, I. *et al.* Hysteresis-Free 1D Network Mixed Halide-Perovskite Semitransparent Solar Cells. *Small* **14**, 1802319 (2018).
7. Marongiu, D. *et al.* Bifacial Diffuse Absorptance of Semitransparent Microstructured Perovskite Solar Cells. *ACS Appl. Mater. Interfaces* **11**, 10021-10027 (2019).
8. Lee, K. *et al.* Neutral-Colored Transparent Crystalline Silicon Photovoltaics. *Joule* **4**, 235-246 (2020).
9. Bechinger, C., Ferrere, S., Zaban, A., Sprague, J. & Gregg, B. A. Photoelectrochromic windows and displays. *Nature* **383**, 608-610, (1996).
10. Wu, J.-J., Hsieh, M.-D., Liao, W.-P., Wu, W.-T. & Chen, J.-S. Fast-Switching Photovoltachromic Cells with Tunable Transmittance. *ACS Nano* **3**, 2297-2303, (2009).
11. Cannavale, A. et al. Photovoltachromic Device with a Micropatterned Bifunctional Counter Electrode. *ACS Appl. Mater. Interfaces* **6**, 2415-2422, (2014).
12. Cannavale, A. et al. Perovskite photovoltachromic cells for building integration. *Energy Environ. Sci.* **8**, 1578-1584, (2015).
13. Zhou, F. et al. Perovskite Photovoltachromic Supercapacitor with All-Transparent Electrodes. *ACS Nano* **10**, 5900-5908, (2016).
14. Xia, X. et al. Perovskite solar cell powered electrochromic batteries for smart windows. *Mater. Horiz.* **3**, 588-595, (2016).
15. Davy, N. C. et al. Pairing of near-ultraviolet solar cells with electrochromic windows for smart management of the solar spectrum. *Nat. Energy* **2**, 17104, (2017).

16. Zhang, D. et al. A Solar-Driven Flexible Electrochromic Supercapacitor. *Materials* **13**, 1206, (2020).

17. Cho, J. et al. Semitransparent Energy-Storing Functional Photovoltaics Monolithically Integrated with Electrochromic Supercapacitors. *Adv. Funct. Mater.* **30**, 1909601, (2020).
