# Supplementary material for: Full-frame and high-contrast smart windows from halide-exchanged perovskites
Source: Nat Commun. 2021 Jun 7;12:3360. doi: 10.1038/s41467-021-23701-z (PMC8184980; doi:10.1038/s41467-021-23701-z)
Supplement: Supplementary file 3 — Description of Additional Supplementary Files [file 41467_2021_23701_MOESM3_ESM.pdf]

Description of Additional Supplementary Files

File Name: Supplementary Movie 1
Description: Solar-Adaptive Monolithic Photovoltachromic Device. The active area of the Photovoltachromic Device is 2 cm × 2 cm.
